# Blood finds a way: pictorial review of thoracic collateral vessels

**DOI:** 10.1186/s13244-019-0753-3

**Published:** 2019-06-13

**Authors:** Thomas J. Marini, Komal Chughtai, Zachary Nuffer, Susan K. Hobbs, Katherine Kaproth-Joslin

**Affiliations:** Department of Imaging Sciences, University of Rochester Medical Center, University of Rochester, 601 Elmwood Ave, Box 648, Rochester, NY 14642 United States

**Keywords:** Veins, Collaterals, Superior vena cava, Superior vena cava syndrome, Thrombosis, Obstruction

## Abstract

In the healthy patient, blood returns to the heart via classic venous pathways. Obstruction of any one of these pathways will result in blood flow finding new collateral pathways to return to the heart. Although significant anatomic variation exists and multiple collateral vessels are often present in the same patient, it is a general rule that the collateral pathways formed are a function of the site of venous blockage. Therefore, knowledge of typical collateral vessel systems can provide insight in localizing venous obstruction and characterizing its severity and chronicity. In addition, knowledge of collateral anatomy can be essential in interventional procedural and/or surgical planning, especially when placing catheters in patients with venous blockage. In this pictorial review, we provide a systematic approach to understanding collateral pathways in patients with venous obstruction in the upper body.

## Key points

- Venous obstruction occurs secondary to mass effect, stenosis, and/or thrombosis.
- No matter the site of obstruction, blood always finds a way back to the heart via collaterals.
- The pattern of collateral vessels that develop is a function of the site of obstruction.
- Axillary and subclavian venous obstruction form collaterals to the ipsilateral shoulder and neck.
- SVC collaterals form as a function of the obstruction’s position relative to the azygos vein.

## Introduction

**Fig. 1:**
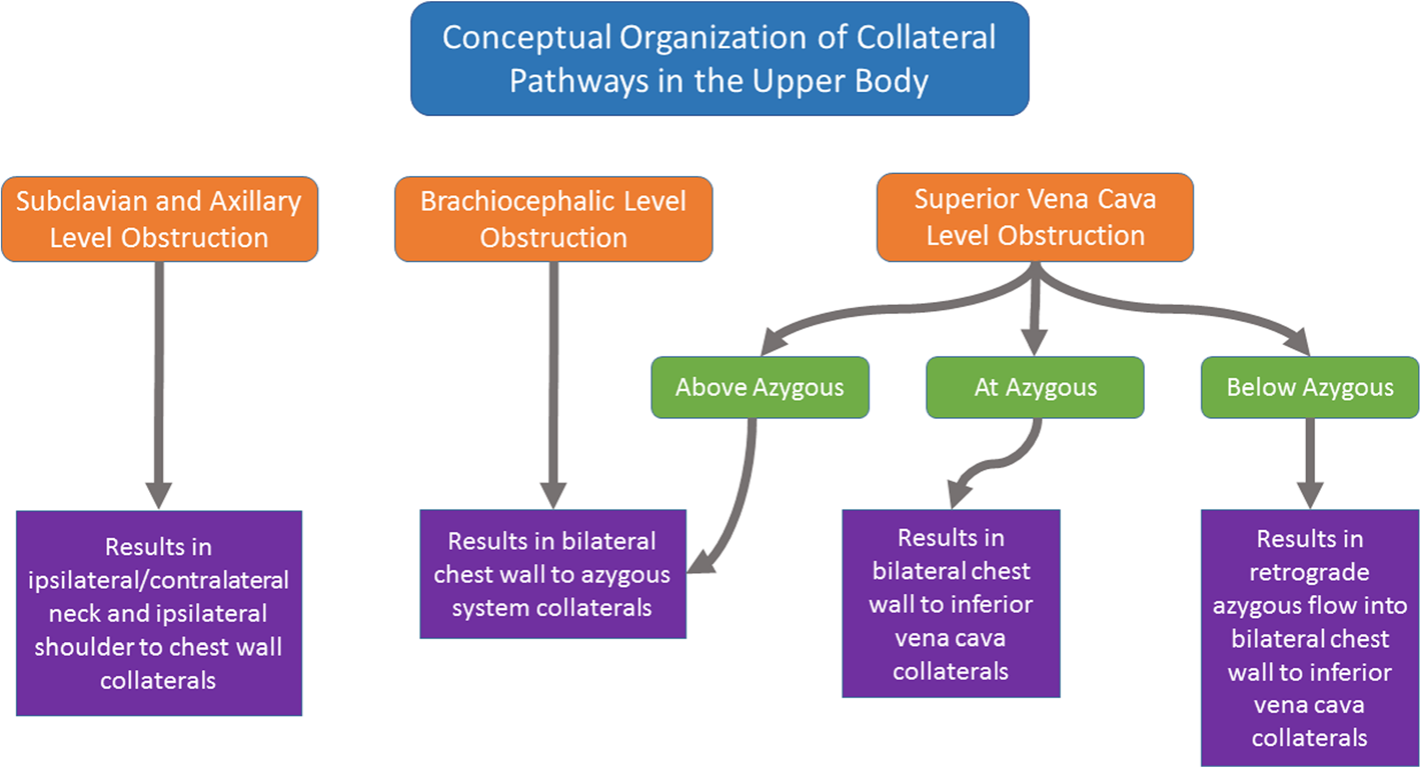
Our conceptual organization of collateral pathways in the upper body

No matter where venous vessel obstruction occurs, blood will try to find a way back to the heart. This typically occurs via changes in direction and/or amount of flow in preexisting venous collateral flow pathways. Despite significant anatomic variation between patients, the pattern and extent of collaterals vessels provide clues to the location, severity, and chronicity of the obstruction. For example, a chronic and complete blockage of the axillary vein would be expected to produce significantly more shoulder collaterals than a partial blockage of the same vessel that developed over a shorter timeframe. Therefore, an understanding of the most common venous collateral pathways can provide the insight necessary for accurate interpretation of imaging studies. In addition, this understanding is important when an intervention is needed, especially when placing central venous catheters. In this pictorial review, we take a systematic approach in using patterns of collaterals to localize the source of obstruction in the venous system of the upper body (Fig. 1).

**Fig. 2:**
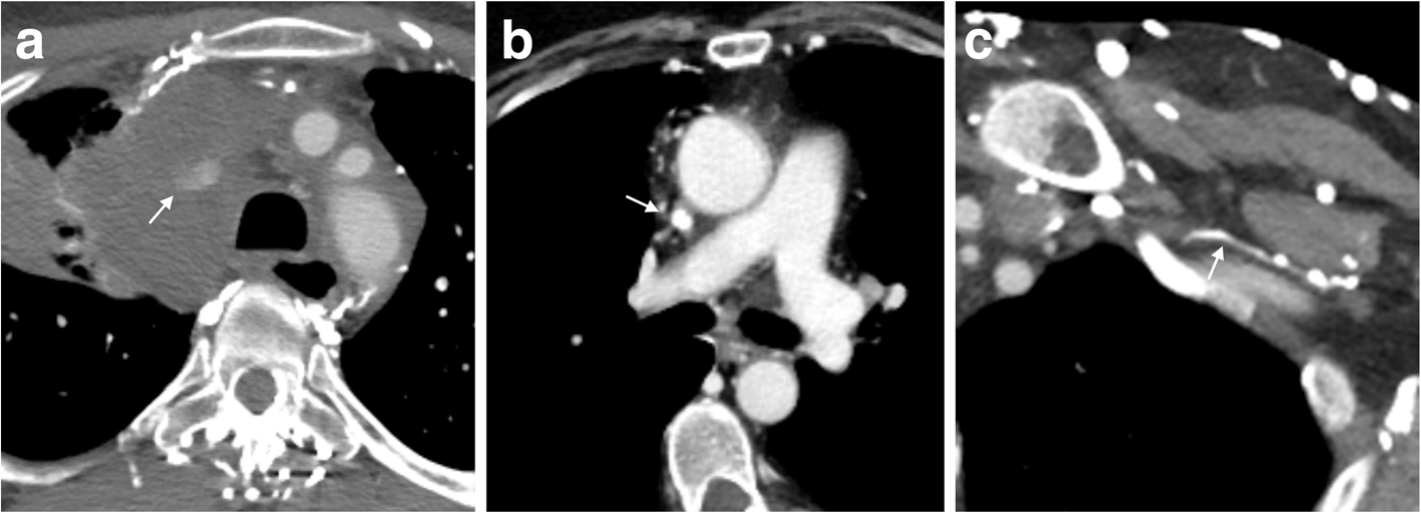
Causes of obstruction. **a** Axial CT image showing complete obstruction of the SVC secondary to extrinsic mass compression (arrow). **b** Axial CT image demonstrating SVC narrowing/occlusion (arrow) secondary to chronic indwelling catheter. **c** Axial CT image demonstrating intraluminal obstruction of the left axillary vein from a chronic thrombus (arrow)

Clinically, patients with venous blockages often present with swelling prior to the site of blockage. Causes of obstruction are varied but can be grouped into three categories of pathology: extrinsic mass effect, narrowing/stenosis, and intraluminal occlusion/thrombosis (Fig. 2). In the chest, mass effect via mediastinal or pulmonary malignancy can physically block venous flow. Superior vena cava (SVC) syndrome is the prototypical example of mass effect resulting in venous obstruction and is well described in the literature [1]. Stenosis or luminal narrowing can develop secondary to radiation, chronic exposure to indwelling catheters, or sequela of prior thrombosis [2]. Finally, intraluminal occlusion can be a product of trauma, hypercoagulable state leading to acute thrombus, tumor, or an indwelling catheter.

## Normal anatomy

**Fig. 3:**
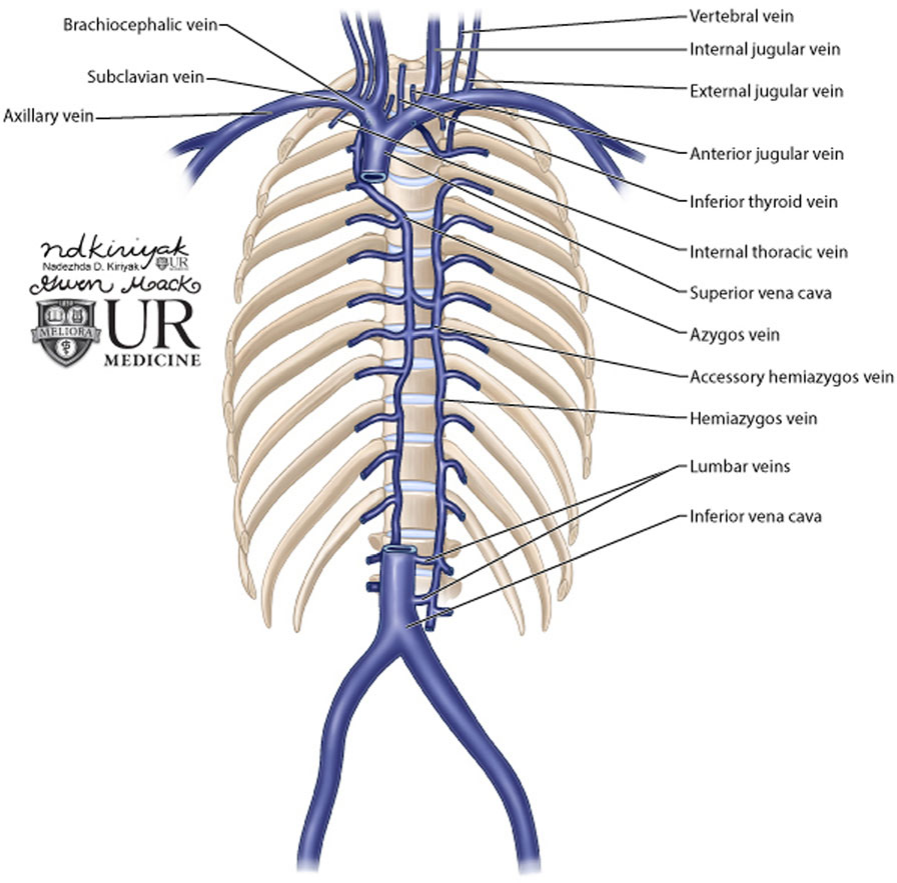
Review of venous anatomy

In normal anatomy, the veins of the arm (cephalic, basilic, and brachial veins) all drain into the axillary vein. At the outer border of the first rib, the axillary vein becomes the subclavian vein. The subclavian vein continues on to receive drainage from the neck. Once the subclavian vein merges with the internal jugular vein, the brachiocephalic vein is formed. The brachiocephalic vein subsequently drains into the SVC. Figure 3 shows an overview of the normal venous anatomy in the upper extremities, neck, and chest [3].

The azygos vein is an otherwise unassuming vein which drains the intercostal system into the lower SVC. However, the importance of the azygos vein in relation to the site of obstruction in these thoracic collateral systems cannot be overstressed. Conceptually, it is important to note that the azygos system can potentially connect to the entire venous supply of the body. Blockages in one part of the thorax tend to divert at least some of the flow into the azygos system where it is subsequently rerouted to bypass the blockage. Any blockage above the azygos vein may be rerouted into the SVC through the azygos system. However, when the SVC is blocked at the level of the azygos, blood may only enter the heart through the inferior vena cava (IVC).

## Overview of collateral imaging

Characterization of collateral vessels is not a common primary indication for imaging, and collaterals are often first identified incidentally or after a complication has occurred. CT angiography is the most commonly used imaging modality to evaluate collateral pathways. A venous phase of imaging can be obtained by performing the diagnostic portion of the scan at 60–90 s post a standard weight-based dose of intravascular contrast administration. Benefits of this imaging modality include its superior spatial resolution with the ability to view images using multiplanar reformats and its ability to image during different phases of contrast enhancement (venous versus arterial), especially as collateral vessels can be difficult to appreciate in the absence of contrast. It is important to note the side of contrast injection may affect the ability to visualize the obstruction, as the collateral flow may only be visualized if injected on the ipsilateral side. Use of diluted contrast during injection can reduce streak artifact during initial phase imaging which may otherwise limit visualization of an area of obstruction or collateral pathways. Drawbacks to this imaging modality include its radiation exposure, its need for intravenous contrast, and its limited enhancement of the veins.

**Fig. 4:**
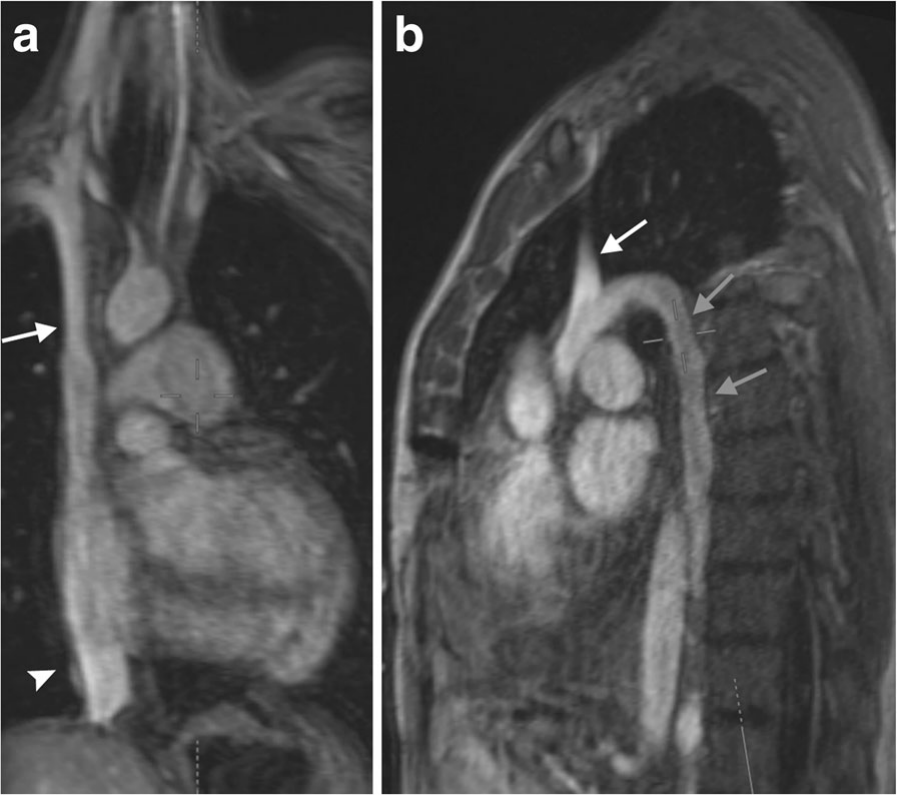
MRI venous imaging. Coronal (**a**) and sagittal (**b**) images of the thoracic venous anatomy in the late arteriovenous phase of imaging of a MRI scan. The SVC (white arrow), IVC (arrowhead), and azygos vein (gray arrows) are labeled

Magnetic resonance venography can be considered superior to CT venography in its evaluation of the deep veins due to its superior contrast side effect profile and lack of radiation exposure (Fig. 4) [4, 5]. Three-dimensional (3D) gadolinium-enhanced gradient echo (GRE) images create a dynamic view of the vessels with several acquisitions acquired sequentially, typically beginning in the arterial phase. If needed for diagnostic interpretation, a selective venous study can be created by subtracting the early arterial phase study from the subsequently obtained mixed venous-arterial phase study. This volumetric approach creates high-resolution and high signal-to-noise images which can be then be reconstructed in any imaging plane.

Digital subtraction angiography in interventional radiology remains the gold standard for characterization of collateral pathways with venograms indicated to confirm suspected venous blockages and provide real-time information on collateral filling. The main drawback to this modality is that it does not provide a detailed look at what is happening extravascularly, leading to an incomplete picture in the evaluation of the blockage.

Finally, venous Doppler ultrasonography is an inexpensive readily available technique that is well tolerated by patients and has no radiation or contrast exposure which can assist in evaluation of venous pathways. Venous ultrasonography can be limited by unreachable anatomy, poor acoustic windows, patient body habitus, and skill of the sonographer [6, 7].

## Axillary and subclavian obstruction

In the past, axillary and subclavian obstructions were relatively rare. However, the incidence of such obstructions has increased proportionately with the increasing frequency of indwelling subclavian catheter use [8]. Axillary and subclavian obstruction results in collaterals to the shoulder and neck.

### Shoulder collaterals

**Fig. 5:**
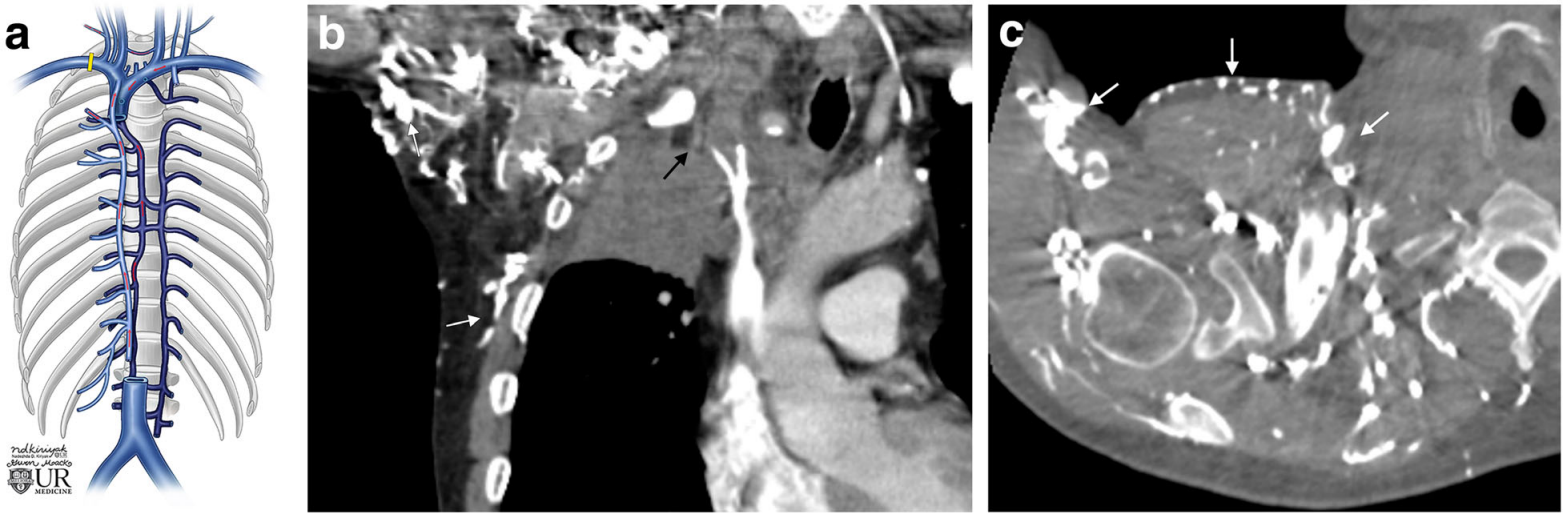
Shoulder/chest wall collaterals. **a** Schematic image of a subclavian obstruction and pathway for collateral flow. **b** Coronal CT image in a 53-year-old patient with a right apical lung mass (stage IV lung adenocarcinoma) obstructing the subclavian venous flow (black arrow) due to mass effect. Extensive shoulder and chest wall collateral formation (white arrows). **c** Axial CT image in the same patient shows extensive unnamed shoulder and chest wall collaterals (white arrows)

Axillary and subclavian blockage most frequently results in collateral pathways to the ipsilateral shoulder (Fig. 5). Shoulder collaterals may involve the lateral thoracic vein, subscapular vein, suprascapular vein, and intercostal veins. Shoulder collaterals can then bypass the blockage directly back into the subclavian and axillary system or drain into the azygos system via the chest wall. Specifically, the costoaxillary veins connect the first to seventh intercostal veins to the lateral thoracic vein.

### Neck collaterals

**Fig. 6:**
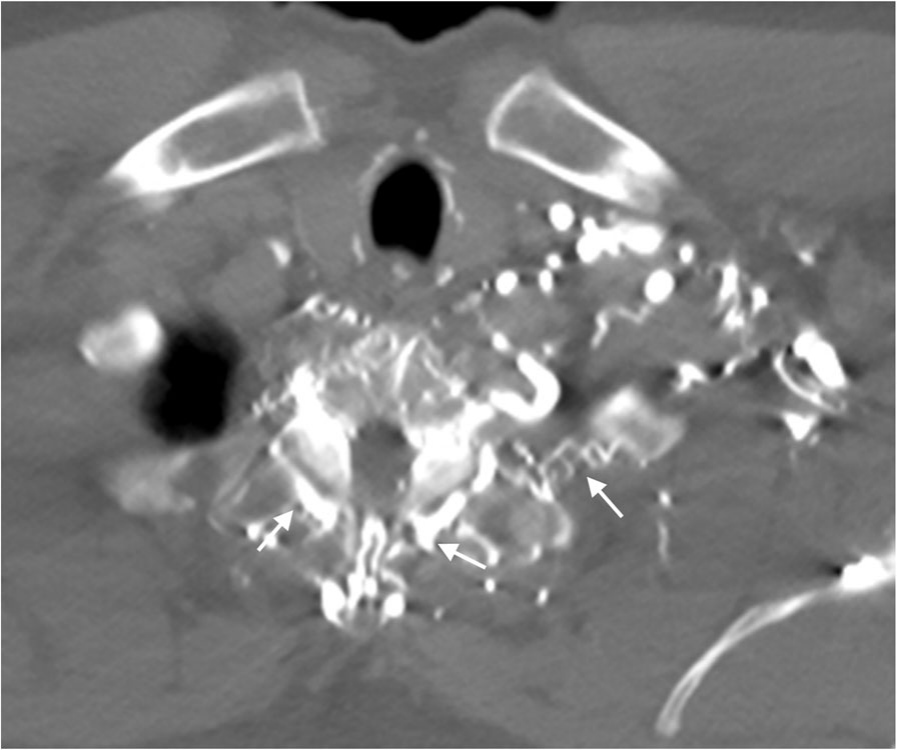
Posterior neck collaterals. Axial CT image showing extensive unnamed vertebral collaterals (arrows) in a patient with occlusion of the left brachiocephalic vein

**Fig. 7:**
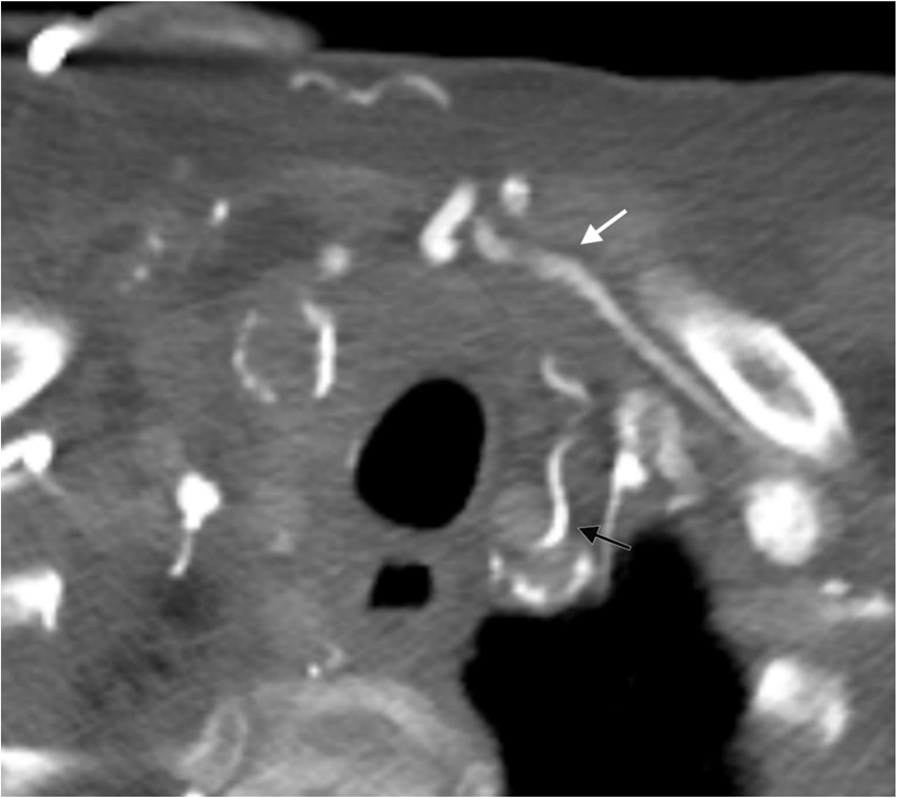
Anterior neck collaterals. Axial CT image showing the jugular venous arch (white arrow) assisting in the diversion of blood flow to the patent contralateral side and an enlarged inferior thyroid vein collateral (black arrow) in a patient with an obstructed internal jugular vein

Collateral pathways also can form to both the anterior and posterior ipsilateral neck veins. Posterior neck collaterals are more common than anterior neck collaterals and are generally more pronounced (Fig. 6). These posterior collaterals can either cross the neck contralaterally or bypass the blockage ipsilaterally. They may also drain directly into the chest wall and the azygos system to return to the SVC. With anterior neck collaterals, blood flow commonly crosses over to the patent contralateral neck (Fig. 7). Vessels involved in the anterior neck collaterals include the vertebral vein, internal jugular vein, external jugular vein, jugular venous arch, and inferior thyroid vein.

## Brachiocephalic obstruction

**Fig. 8:**
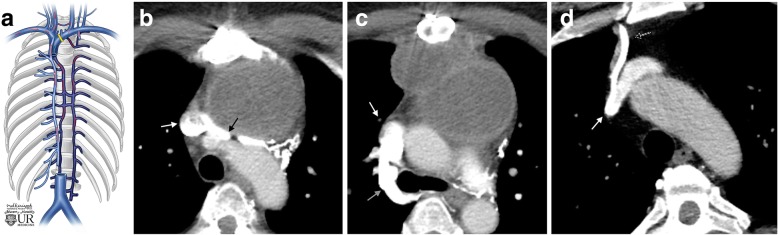
Brachiocephalic blockage. **a** Schematic image of a brachiocephalic obstruction and pathway for collateral flow. **b** Axial CT image of a 59-year-old female with a mediastinal tumor demonstrating extrinsic compression of the left brachiocephalic vein (black arrow) before it enters the SVC (white arrow). **c** Axial CT image in the same patient showing a dilated azygos vein (gray arrow) draining into the SVC (white arrow). **d** Axial CT image in a different patient demonstrates a prominent right internal thoracic vein (dashed arrow) draining into the SVC (white arrow) bypassing a right-sided brachiocephalic blockage (not pictured)

As will be seen with SVC obstruction above the level of the azygos vein, blockages that occur in the brachiocephalic vein will funnel blood flow through the azygos collateral system and azygos vein into the inferior SVC. Brachiocephalic blockages commonly have internal thoracic vein collaterals flow with the direction of flow dependent on the location of the blockage. If the blockage occurs upstream of the origin of the internal thoracic vein, the internal thoracic vein returns blood to the downstream brachiocephalic vein via antegrade flow. If the blockage occurs downstream of the origin of the internal thoracic vein, flow reverses in the internal thoracic vein and flow will return to the SVC via the azygos system (Fig. 8).

## Superior vena cava obstruction

SVC obstruction presents a substantial physiological challenge as it involves the final pathway for venous return of the upper body. Three patterns of collateral vessel formation are observed in SVC obstruction, all resulting from the location of the blockage relative to the azygos vein [9, 10]. As detailed in the anatomy section, the azygos vein delivers venous return to the inferior SVC and is the only major vein to feed into the SVC apart from the right and left brachiocephalic veins. As the azygos system functionally connects in some way to the entire venous system, its importance in bypassing SVC obstruction is not surprising.

### Blockages above the azygos

**Fig. 9:**
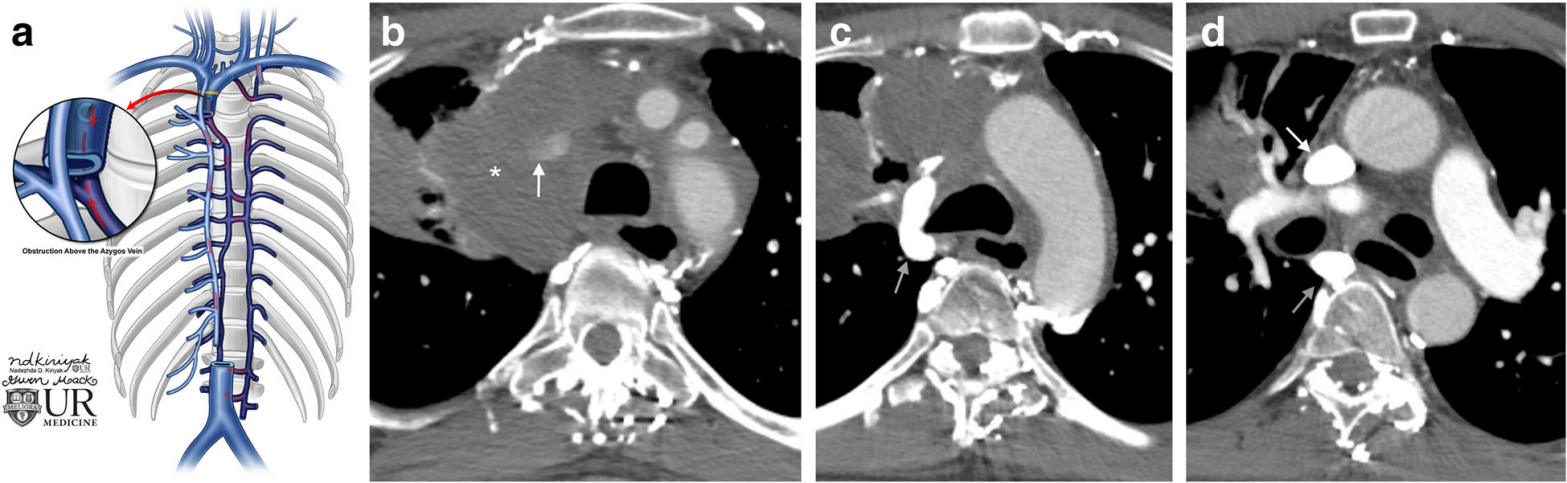
Blockage of the SVC above the azygos vein. **a** Schematic image of a SVC obstruction above the level of the azygos vein and pathway for collateral flow. **b** Axial CT image of a 46-year-old male with squamous cell carcinoma (asterisk) causing SVC compression (white arrow) above the azygos vein due to extrinsic mass effect. **c** Axial CT image showing a dilated azygos vein (gray arrow) entering into the SVC below the blockage. **d** Axial CT image shows contrast enhancement of the SVC downstream of the blockage (white arrow); the dilated azygos vein is also seen (gray arrow)

A blockage upstream of the azygos vein means that blood can reenter the SVC below the level of blockage via a patent azygos vein. Collaterals upstream of the obstruction will form to the azygos system and intercostal veins, funneling the blood flow back into the downstream SVC via normal forward flow through the azygos vein (Fig. 9).

### Blockages at the level of the azygos

**Fig. 10:**
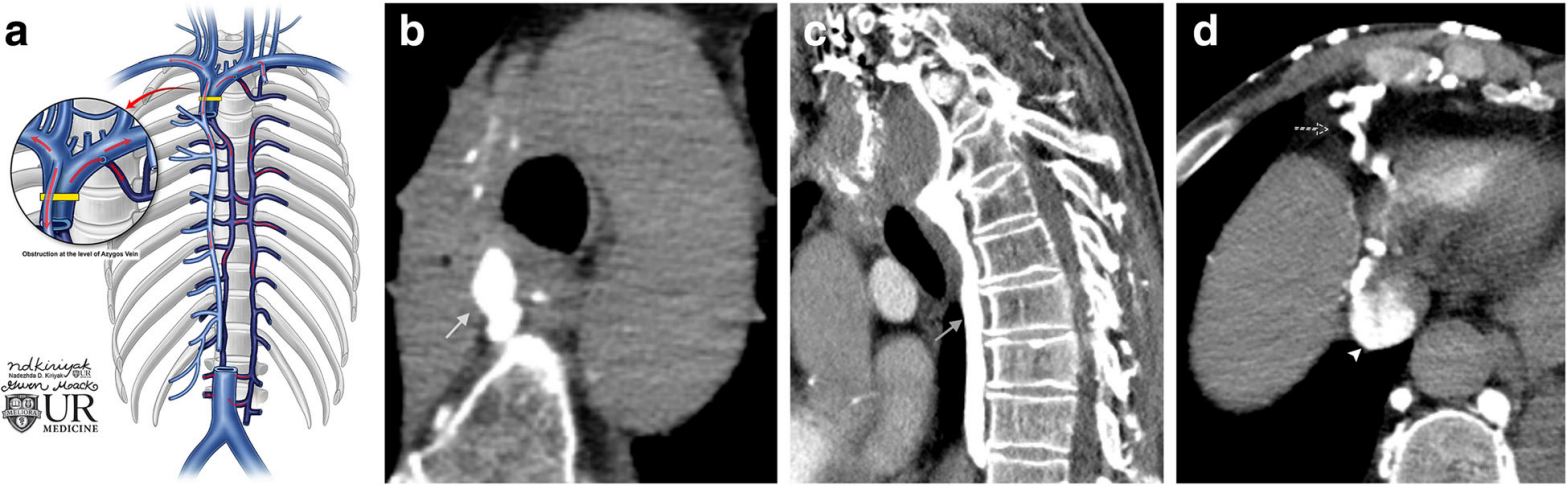
Blockage of the SVC at the level of the azygos vein. **a** Schematic image of a SVC obstruction at the level of the azygos vein and pathway for collateral flow. **b** Axial CT image in a patient with a right hilar mass at the level of the SVC/azygous junction, causing the azygos vein (gray arrow) to become dilated. **c** Coronal CT image in the same patient demonstrating a prominent azygos vein (gray arrow) shunting blood inferiorly toward the IVC. **d** Blood from the azygos system in this patient is eventually shunted through collateral vessels (dashed arrow) into the IVC (arrowhead) bypassing the SVC blockage at the level of the azygos

If the SVC blockage is at the level of the azygos vein, then there is no physiological pathway for blood to reenter the SVC. Blood must reverse flow in the azygos system into the lower venous system and the inferior vena cava (IVC) (Fig. 10). Many different potential collateral pathways exist for the blood to enter the IVC. Hepatic collaterals (draining into the IVC) and collateralized lumbar veins (connecting to the common iliac veins and IVC) are two common examples by which blood is shunted via reverse flow to the patent IVC.

### Blockages below the azygos

**Fig. 11:**
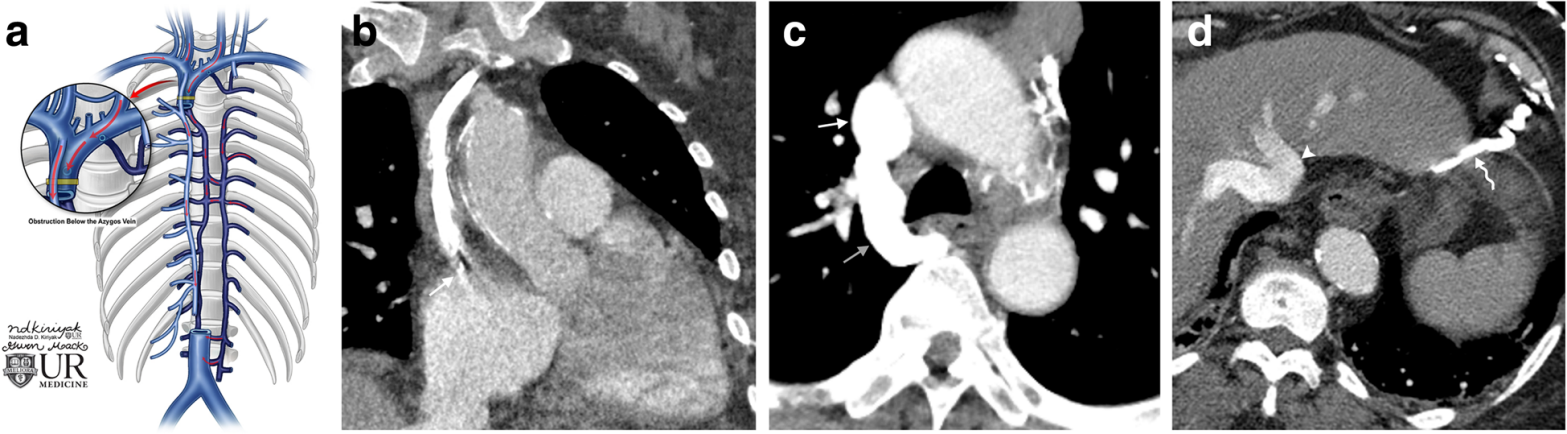
Blockage of the SVC below the azygos vein. **a** Schematic image of a SVC obstruction at the level of the azygos vein and pathway for collateral flow. **b** Coronal CT image shows occlusion of the SVC below the level of the azygos vein (white arrow). **c** Axial CT image demonstrates retrograde contrast flow through the azygos vein (gray arrow) from the upper SVC (white arrow). **d** Axial CT image showing diaphragmatic collaterals (curved arrow) draining to the IVC (arrowhead) bypassing the inferior SVC blockage below the level of the azygos vein

Blockages below the azygos prevent any flow of blood to the heart via the SVC. However, in this case, blood will be redirected into the azygous vein and azygous system in a retrograde manner (Fig. 11). Ultimately, the blood will form collateral vessels to the IVC as discussed in the previous category of blockage at the level of the azygos vein.

## Unusual collaterals

**Fig. 12:**
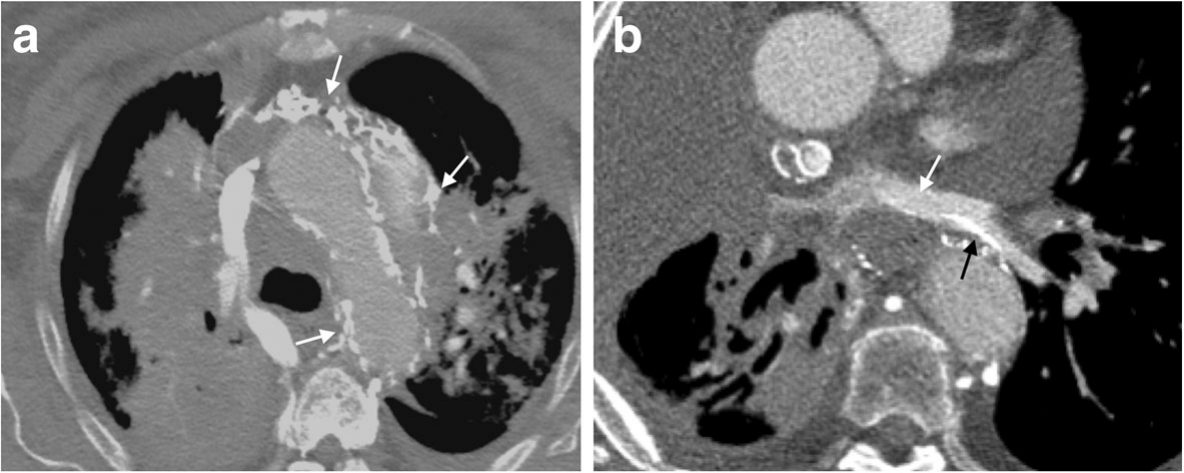
Unusual collaterals. **a** Axial CT image of a patient with numerous unnamed collateral vessels coursing through the mediastinum (white arrows). **b** Axial CT scan demonstrates collateral vessels (black arrow) draining into the left pulmonary veins (white arrow)

It is important to note that we have presented common collateral pathways in this pictorial review. In reality, collateral pathways demonstrate a great deal of anatomical variation. Furthermore, uncommon collateral pathways involving the mediastinum and pulmonary vasculature (among others) have been described and should also be appreciated if present (Fig. 12) [11]. These pathways are often a sign of serious pathology and warrant immediate search for an underlying cause.

## Collateral placement of lines

**Fig. 13:**
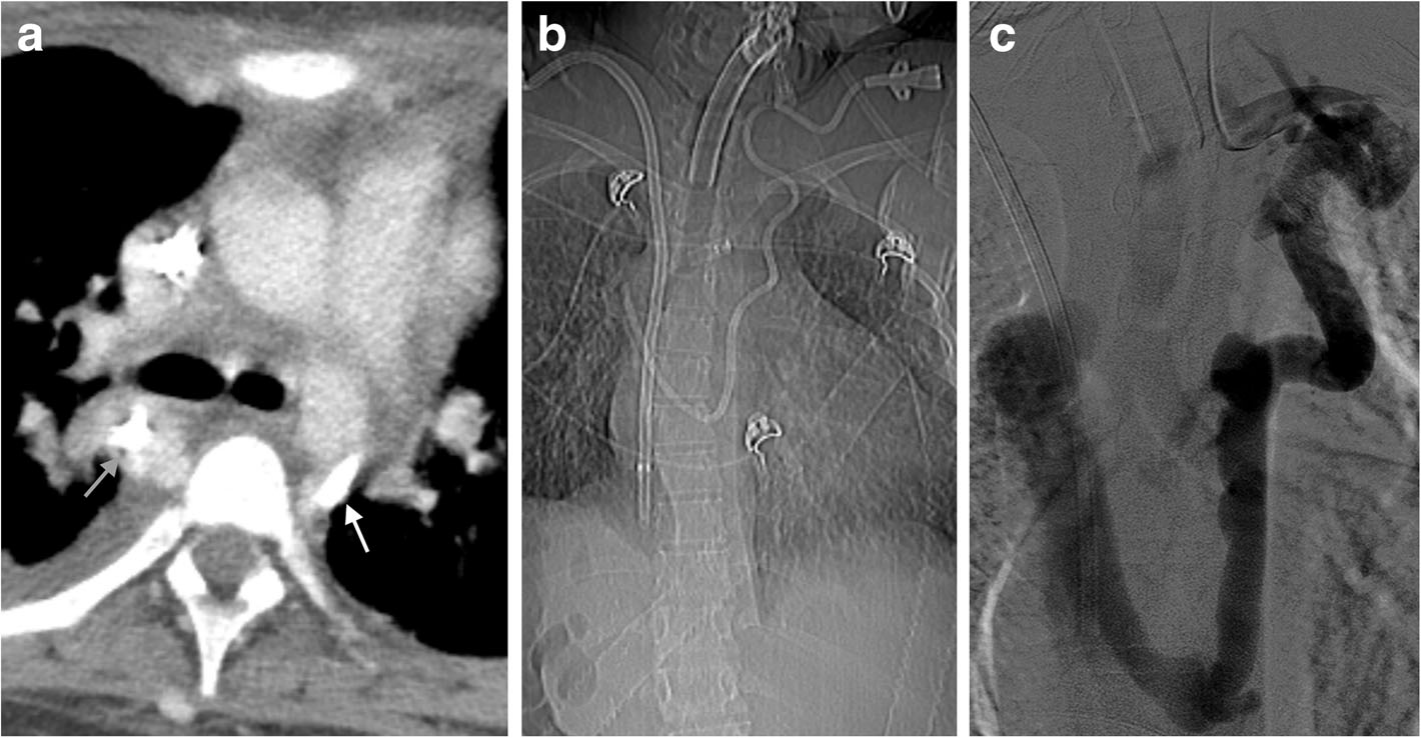
Collateral placement of central lines. **a** Axial CT image demonstrating placement of a central line through the hemiazygous (white arrow) and azygos vein (gray arrow). **b** CT scout image showing the tortuous path the line takes through the collateral vessels to the right atrium. **c** Interventional venogram of the same patient demonstrating collateral placement of the line

Collateral anatomy is of particular importance in interventional radiology. In some patients with venous obstruction, direct knowledge of collateral vessels may be necessary to place lines (Fig. 13). While collateral vessels are often discovered accidentally, interventional radiologists may also use them intentionally in patients with less than ideal venous access.

## Treatment

**Fig. 14:**
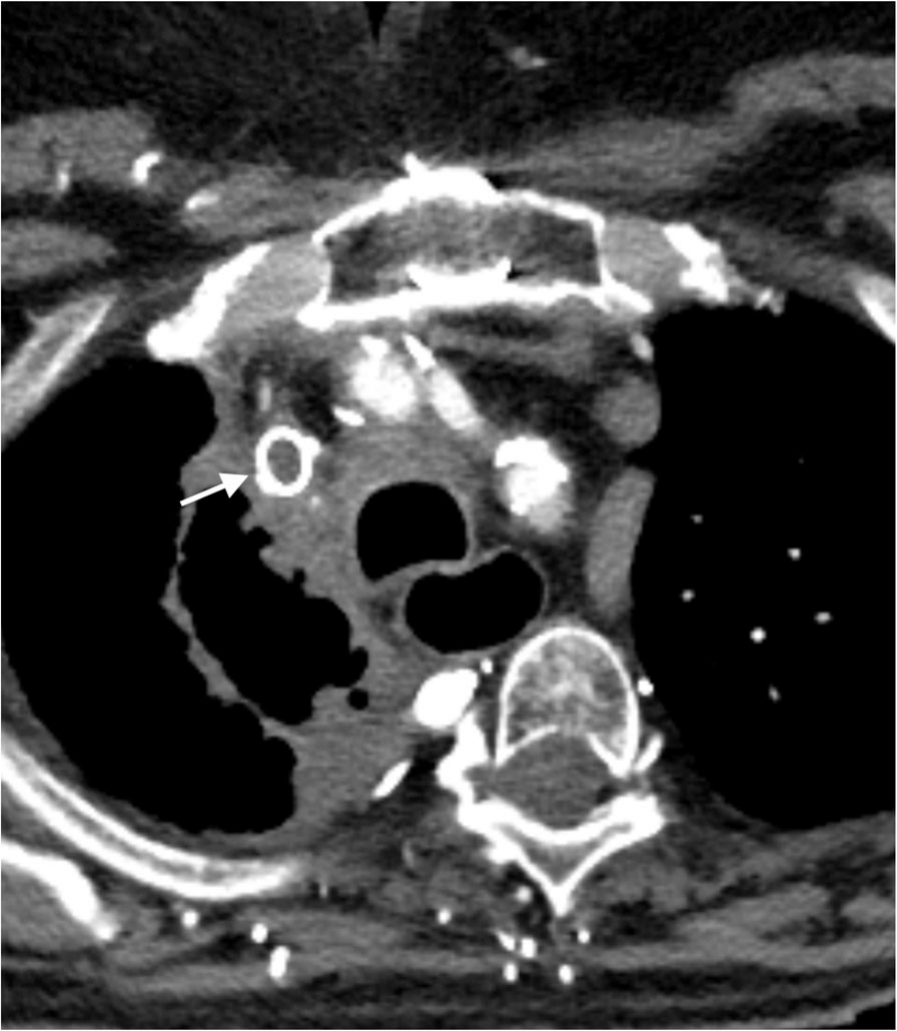
Thrombosed stent. Axial CT image demonstrating a patient who has a thrombosed stent within the SVC (arrow)

Treatment of these conditions involves correcting the underlying factor responsible for the obstruction. In some cases, palliative stenting may be attempted, although this is not always successful (Fig. 14) [12]. Thrombosis of stents is a common complication.

## Summary

Venous obstruction may result from mass effect, stenosis, or thrombosis. No matter the cause, blood always finds a way around blockages to return to the heart. The path blood takes around blockages is predictable depending on the location and size of the obstruction. Thus, by studying collateral vessels, we can gain insight into an obstruction’s location and severity. Axillary and subclavian obstructions form shoulder and neck collaterals that can bypass the blockage. Collaterals secondary to SVC obstruction form in a pattern dependent on the location of the obstruction relative to the azygos vein. It is the role of the radiologist to apply knowledge of these collateral pathways to assist in diagnosis and intervention in patients with venous obstruction.

## Data Availability

Not applicable

## Abbreviations

IVC: Inferior vena cava
SVC: Superior vena cava
